# Supplementary material for: Riboflavin, a Potent Neuroprotective Vitamin: Focus on Flavivirus and Alphavirus Proteases
Source: Microorganisms. 2022 Jun 30;10(7):1331. doi: 10.3390/microorganisms10071331 (PMC9315535; doi:10.3390/microorganisms10071331)
Supplement: Supplementary file 1 [file microorganisms-10-01331-s001.zip › microorganisms-1754607-supplementary.pdf]

Supplementary material

Riboflavin, a Potent Neuroprotective Vitamin: Focus on flavivirus and alphavirus proteases

Raphael J. Eberle [1,2,†,*], Danilo S. Olivier [3], Marcos S. Amaral [4], Carolina C. Pacca[5,6], Mauricio L. Nogueira[6,7], Raghuvir K. Arni[8], Dieter Willbold [1,2,9], Monika A. Coronado [1,†,*]

[1] Institute of Biological Information Processing (IBI-7: Structural Biochemistry), Forschungszentrum Jülich, Jülich, Germany; d.willbold@fz-juelich.de
[2] Institut für Physikalische Biologie, Heinrich-Heine-Universität Düsseldorf, Universitätsstraße, Düsseldorf, Germany.
[3] Center of Integrated Sciences, Campus Cimba, Federal University of Tocantins, Araguaína, TO, Brazil; dolivier@gmail.com
[4] Institute of Physics, Federal University of Mato Grosso do Sul, Campo Grande-MS, Brazil; marcos.amaral@ufms.br
[5] FACERES Medical School, São José do Rio Preto, Brazil; carolpacca@gmail.com
[6] Faculdade de Medicina de São José do Rio Preto-FAMERP, São José do Rio Preto, Brazil; mauricio.nogueira@edu.famerp.br
[7] Department of Pathology, University of Texas Medical Branch, Galveston, TX, USA
[8] Multiuser Center for Biomolecular Innovation, Department of Physics, IBILCE, São Paulo State University. São Jose do Rio Preto, Brazil; raghuvir.arni@unesp.br
[9] JuStruct: Jülich Centre for Structural Biology, Forchungszentrum Jülich, Jülich, Germany.

* Correspondence: m.coronado@fz-juelich.de (M.A.C.); r.eberle@fz-juelich.de (R.J.E.); Tel.: +49 2461 61-9505 (M.A.C. and R.J.E.).
† These authors contributed equally to this work.

Table of contents

Supplementary Figure S1. Molecular structures of riboflavin and the vitamin related cofactors FMN and FAD.
Supplementary Figure S2. SDS PAGE (15%) after expression and purification of ZIKV NS2B/NS3^{pro}, YFV NS2B/NS3^{pro} and CHIKV nsP2^{pro}.
Supplementary Figure S3. Normalized activity and inhibition effect of riboflavin over ZIKV NS2B/NS3^{pro}, YFV NS2B/NS3^{pro} and CHIKV nsP2^{pro}.
Supplementary Figure S4. Thermal denaturation of ZIKV NS2B/NS3^{pro}, YFV NS2B/NS3^{pro} and CHIKV nsP2^{pro}-riboflavin complexes using fluorescence spectroscopy.
Supplementary Figure S5. Clustering analysis of ZIKV NS2B/NS3^{pro}-riboflavin complex after MD simulation.
Supplementary Figure S6. Clustering analysis of YFV NS2B/NS3^{pro}-riboflavin complex after MD simulation.
Supplementary Figure S7. Clustering analysis of CHIKV nsP2^{pro}-riboflavin complex after MD simulation.
Supplementary Figure S8. Time dependent modifications of the ZIKV NS2B/NS3^{pro}-riboflavin complex.
Supplementary Figure S9. Time dependent modifications of the YFV NS2B/NS3^{pro}-riboflavin complex.
Supplementary Figure S10. Time dependent modifications of the CHIKV nsP2^{pro}-riboflavin complex.
Supplementary Figure S11. Decomposition of the binding energy of ZIKV NS2B/NS3^{pro}-riboflavin complex of three independent replicas.
Supplementary Figure S12. Decomposition of the binding energy of YFV NS2B/NS3^{pro}-riboflavin complex of three independent replicas.
Supplementary Figure S13. Decomposition of the binding energy of CHIKV nsP2^{pro}-riboflavin complex of three independent replicas
Supplementary Figure S14. Cytoxicity assay of riboflavin against vero cells.

Supplementary Table S1. Virus protease amino acid residues forming hydrogen bonds and hydrophobic interaction with riboflavin.
Supplementary Table S2. Contributing amino acids in the Substrate-binding sites of ZIKV, YFV NS2B/NS3^{pro} and CHIKV nsP2^{pro}.

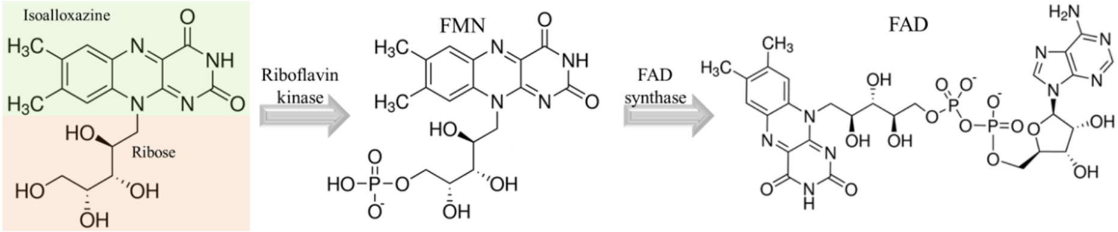

Figure S1. Molecular structures of riboflavin and the vitamin related cofactors FMN and FAD.

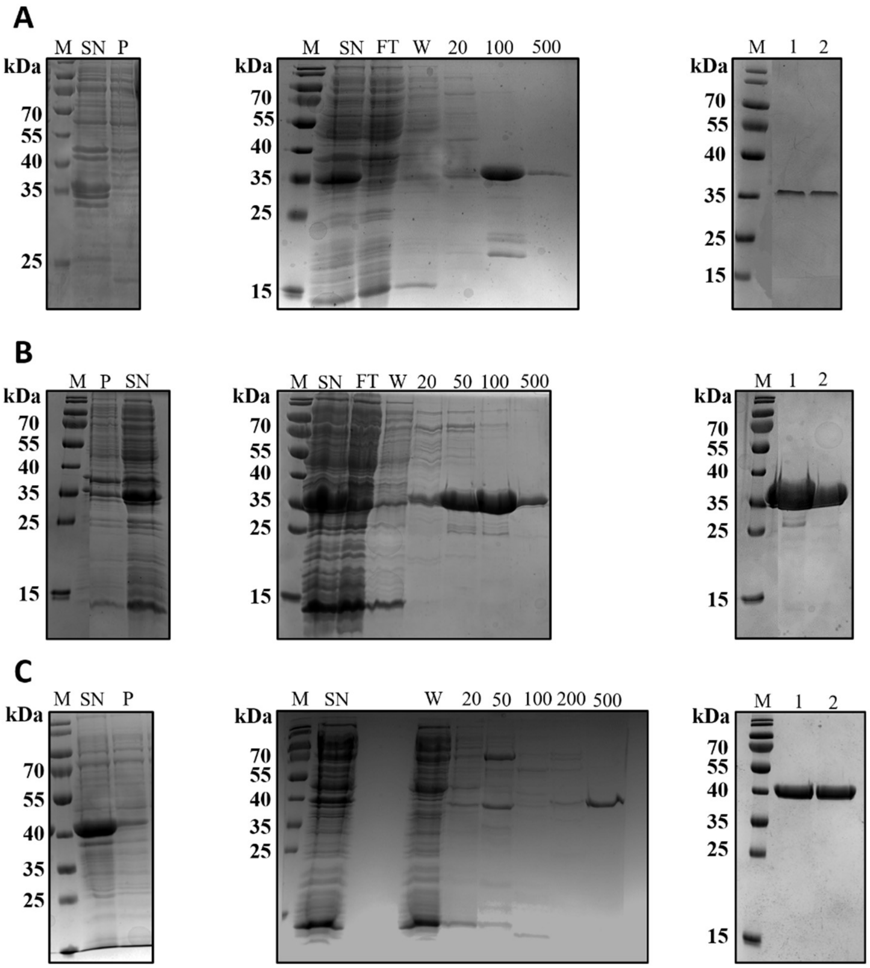

Figure S2. SDS PAGE (15%) after expression and purification of ZIKV NS2B/NS3^{pro}, YFV NS2B/NS3^{pro} and CHIKV nsP2^{pro}. M: Molecular mass marker, SN: supernatant, P: cell pellet, FT: flow through, W: washing step without imidazole, Elution: elution steps with imidazole concentration in mM, 1: protease sample after affinity chromatography and 2: protease sample after size exclusion chromatography. The SDS PAGE 15% demonstrate the purity of the proteases after size exclusion chromatography. **(A)** ZIKV NS2B/NS3^{pro}, **(B)** YFV NS2B/NS3^{pro} and **(C)** CHIKV nsP2^{pro}.

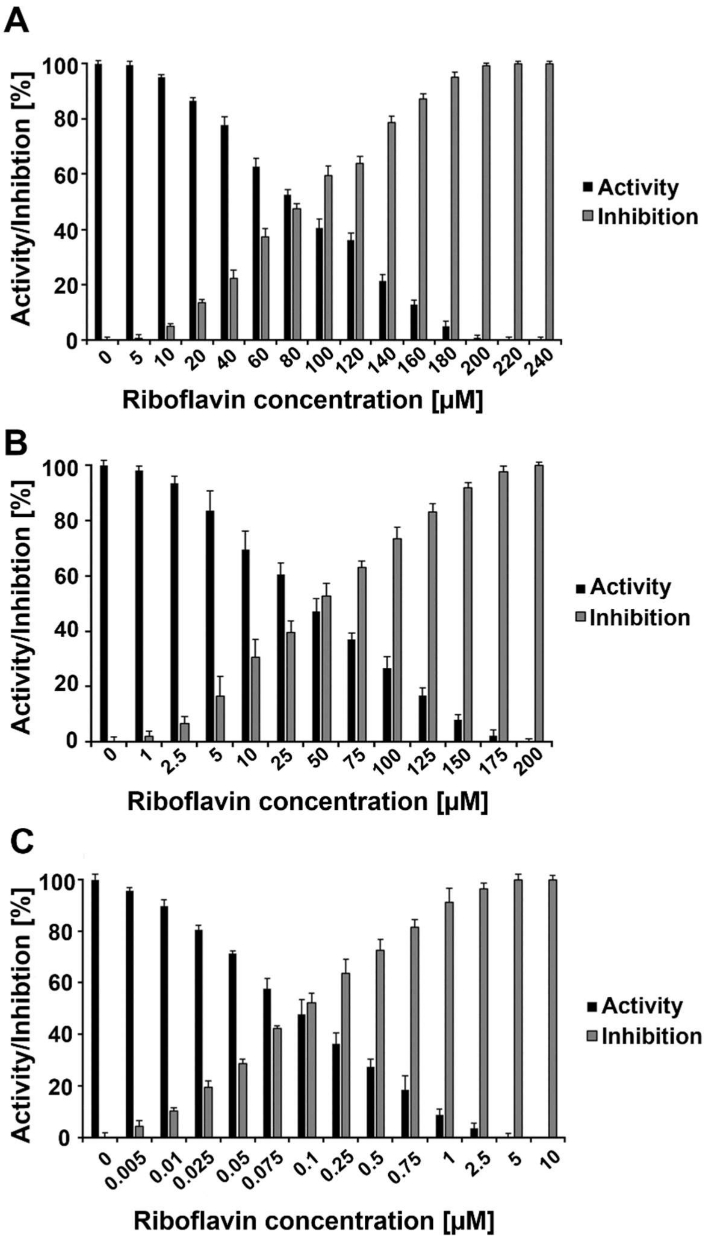

Figure S3. Normalized activity and inhibition effect of riboflavin over ZIKV NS2B/NS3^{pro}, YFV NS2B/NS3^{pro} and CHIKV nsP2^{pro}. Data shown are the mean ± SD from 3 independent measurements (n=3). **(A)** ZIKV NS2B/NS3^{pro} under riboflavin influence. **(B)** YFV NS2B/NS3^{pro} under riboflavin influence. **(C)** CHIKV nsP2^{pro} under riboflavin influence.

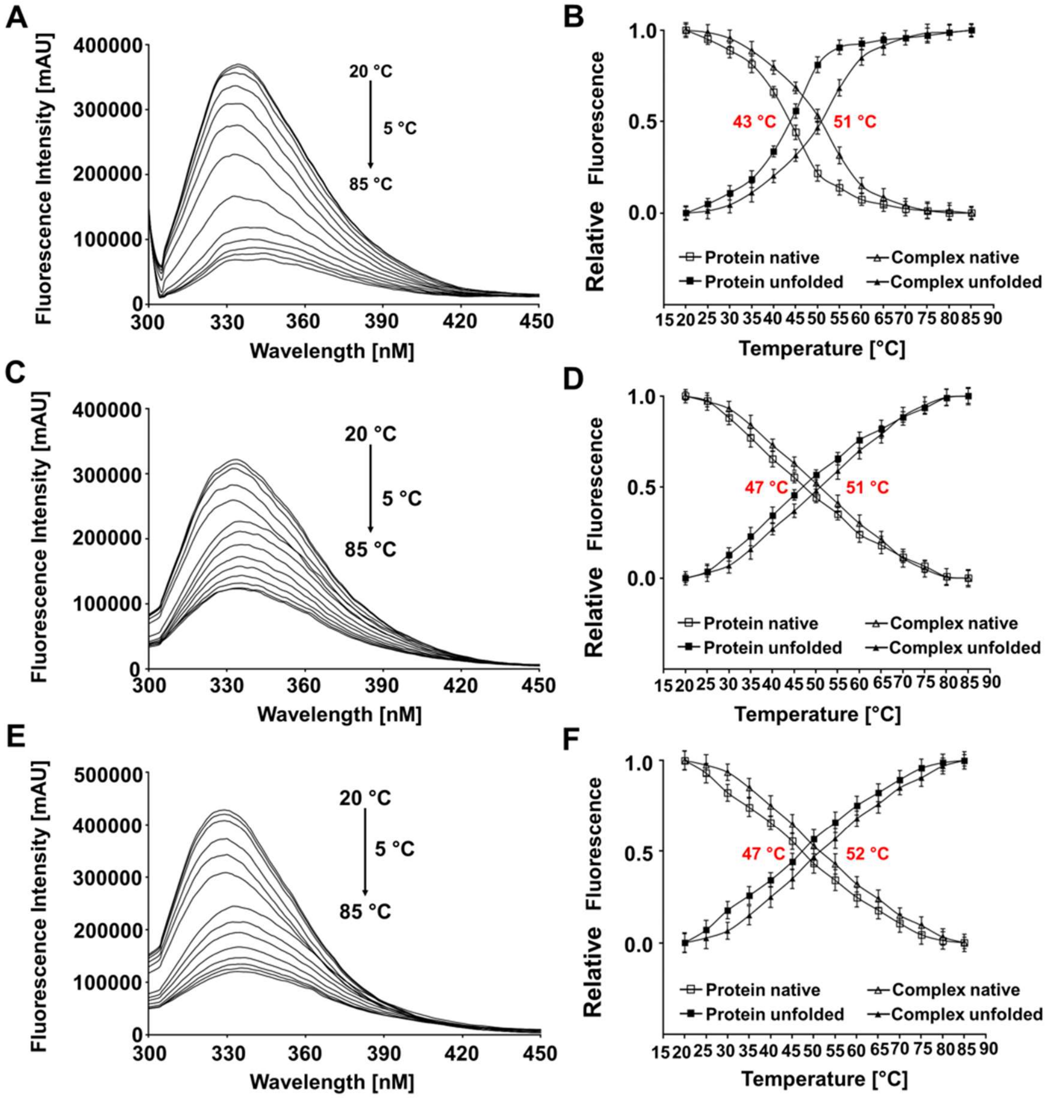

Figure S4. Thermal denaturation of ZIKV NS2B/NS3^{pro}, YFV NS2B/NS3^{pro} and CHIKV nsP2^{pro}-riboflavin complexes using fluorescence spectroscopy. Data shown are the means ± SD from three independent measurements (n=3). **(A)** Fluorescence spectra during thermal denaturation of ZIKV NS2B/NS3^{pro}-riboflavin complex. **(B)** Plot of the native protein fraction (fN) and the unfolding protein fraction (fU), during thermal denaturation from 20 to 85 °C. The melting temperature (T_m) of 43 °C was determined for ZIKV NS2B/NS3^{pro} and for the ZIKV NS2B/NS3^{pro}-riboflavin complex the T_m increased to 51 °C. **(C)** Fluorescence spectra during thermal denaturation of YFV NS2B/NS3^{pro}-riboflavin complex. **(D)** Plot of the native protein fraction (fN) and the unfolding protein fraction (fU), during thermal denaturation from 20 to 85 °C. T_m of 47 °C was determined for YFV NS2B/NS3^{pro} and for the YFV NS2B/NS3^{pro}-riboflavin complex the T_m increased to 51 °C. **(E)** Fluorescence spectra during thermal denaturation of CHIKV nsP2^{pro}-riboflavin complex. **(F)** Plot of the native protein fraction (fN) and the unfolding protein fraction (fU), during thermal denaturation from 20 to 85 °C. T_m of 47 °C was determined for CHIKV nsP2^{pro} and for the CHIKV nsP2^{pro}-riboflavin complex the T_m increased to 52 °C.

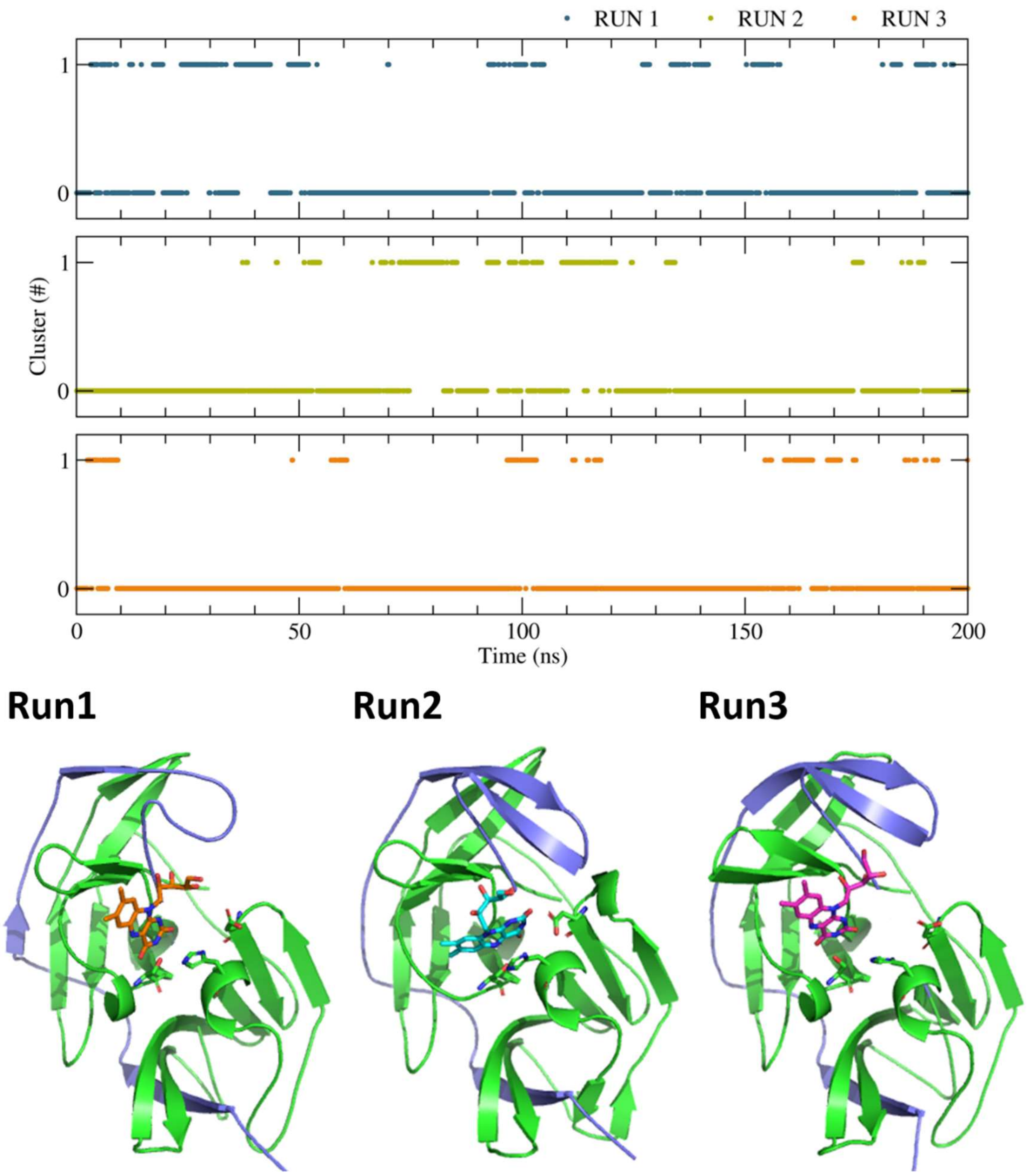

Figure S5. Clustering analysis of ZIKV NS2B/NS3^{pro}-riboflavin complex after MD simulation. Complex structure pointed to a chosen cluster. 3D representation of ZIKV NS2B/NS3^{pro}-riboflavin complex (NS2B/NS3^{pro} in ribbon presentation, NS2B in blue and NS3 in green, active site triad and riboflavin in sticks).

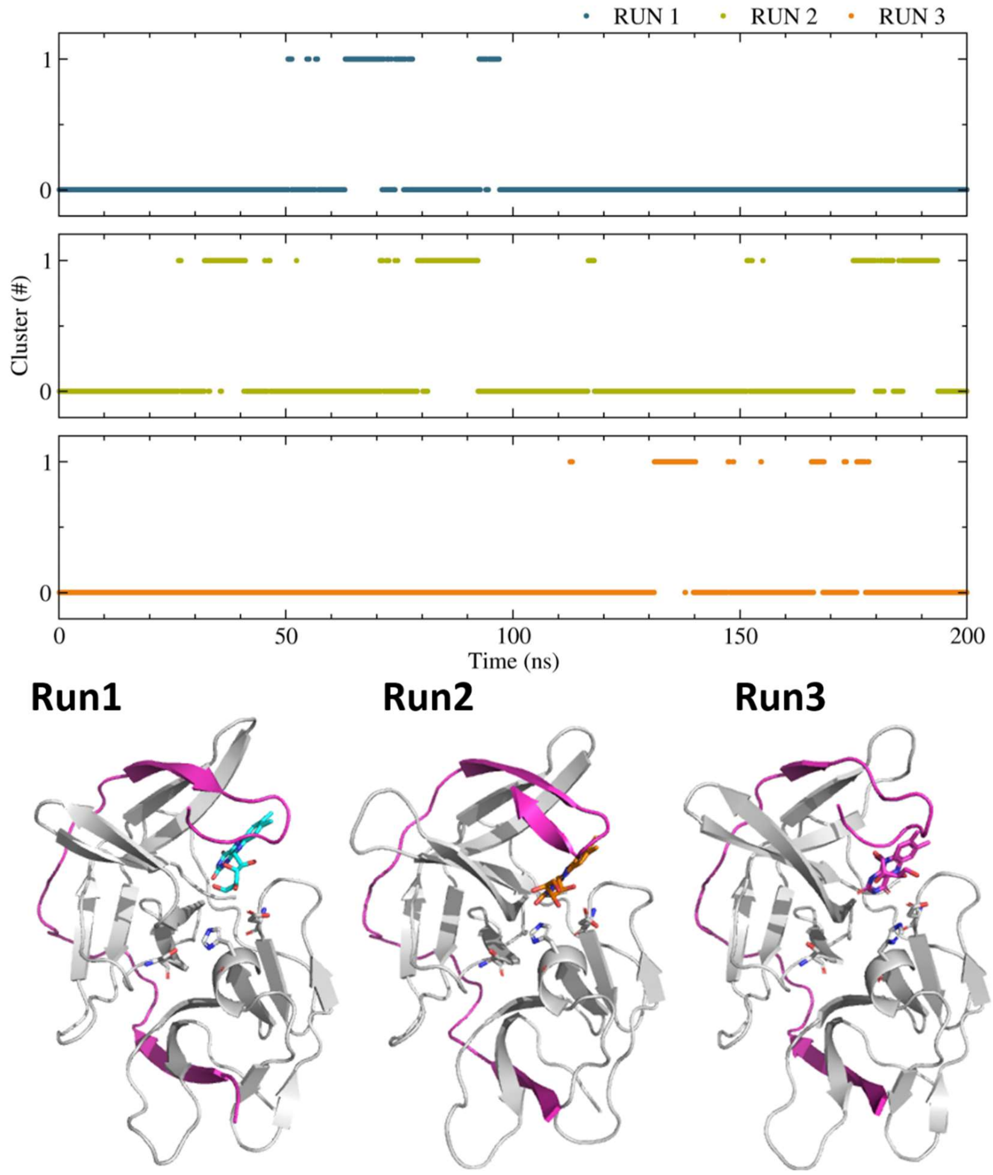

Figure S6. Clustering analysis of YFV NS2B/NS3^{pro}-riboflavin complex after MD simulation. Complex structure pointed to a chosen cluster. 3D representation of YFV NS2B/NS3^{pro}-riboflavin complex (NS2B/NS3^{pro} in ribbon presentation, NS2B in magenta and NS3 in grey, active site triad and riboflavin in sticks).

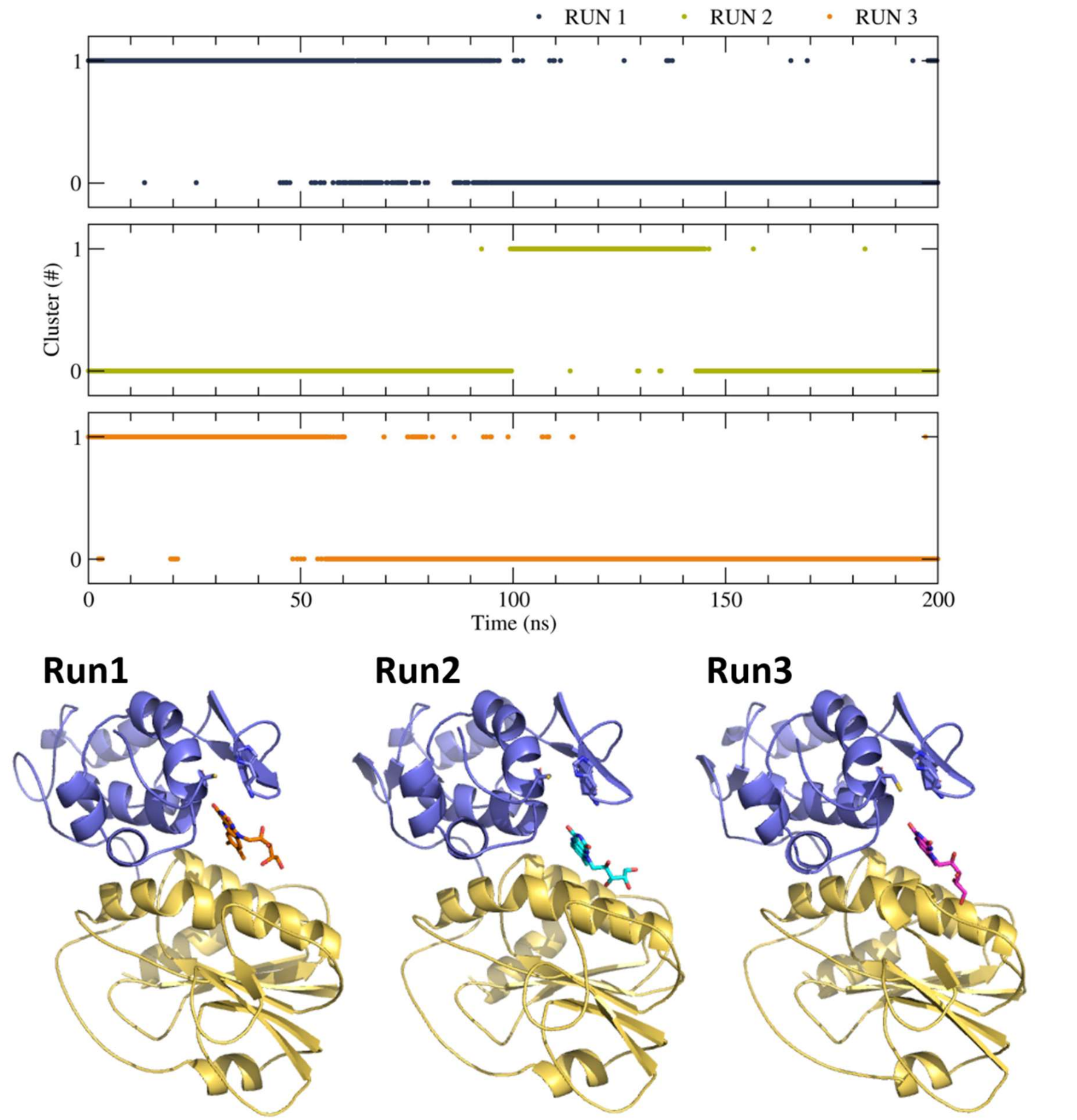

Figure S7. Clustering analysis of CHIKV nsP2^{pro}-riboflavin complex after MD simulation. Complex structure pointed to a chosen cluster. 3D representation of CHIKV nsP2^{pro}-riboflavin complex (nsP2 in ribbon presentation, cysteine protease domain in blue and methyl transferase domain in light yellow, active site dyad and riboflavin in sticks).

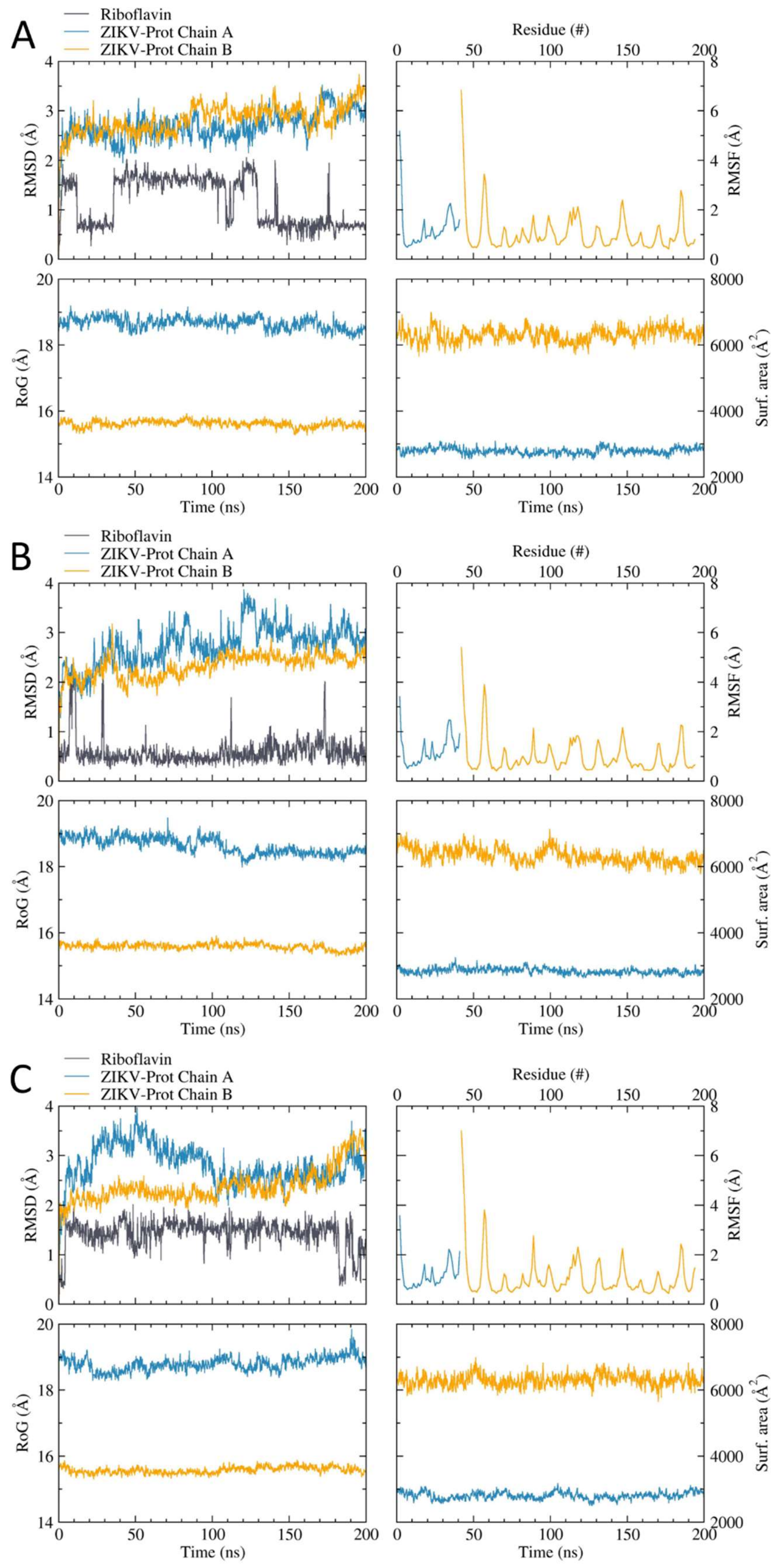

Figure S8. Time dependent modifications of the ZIKV NS2B/NS3^{pro}-riboflavin complex. NS2B (blue), NS3pro (orange) and riboflavin (dark grey). RMSD, RMSF, RoG and surface area as function of time. RMSF for each amino acid. **(A)** replicate one, **(B)** replicate two and **(C)** replicate three.

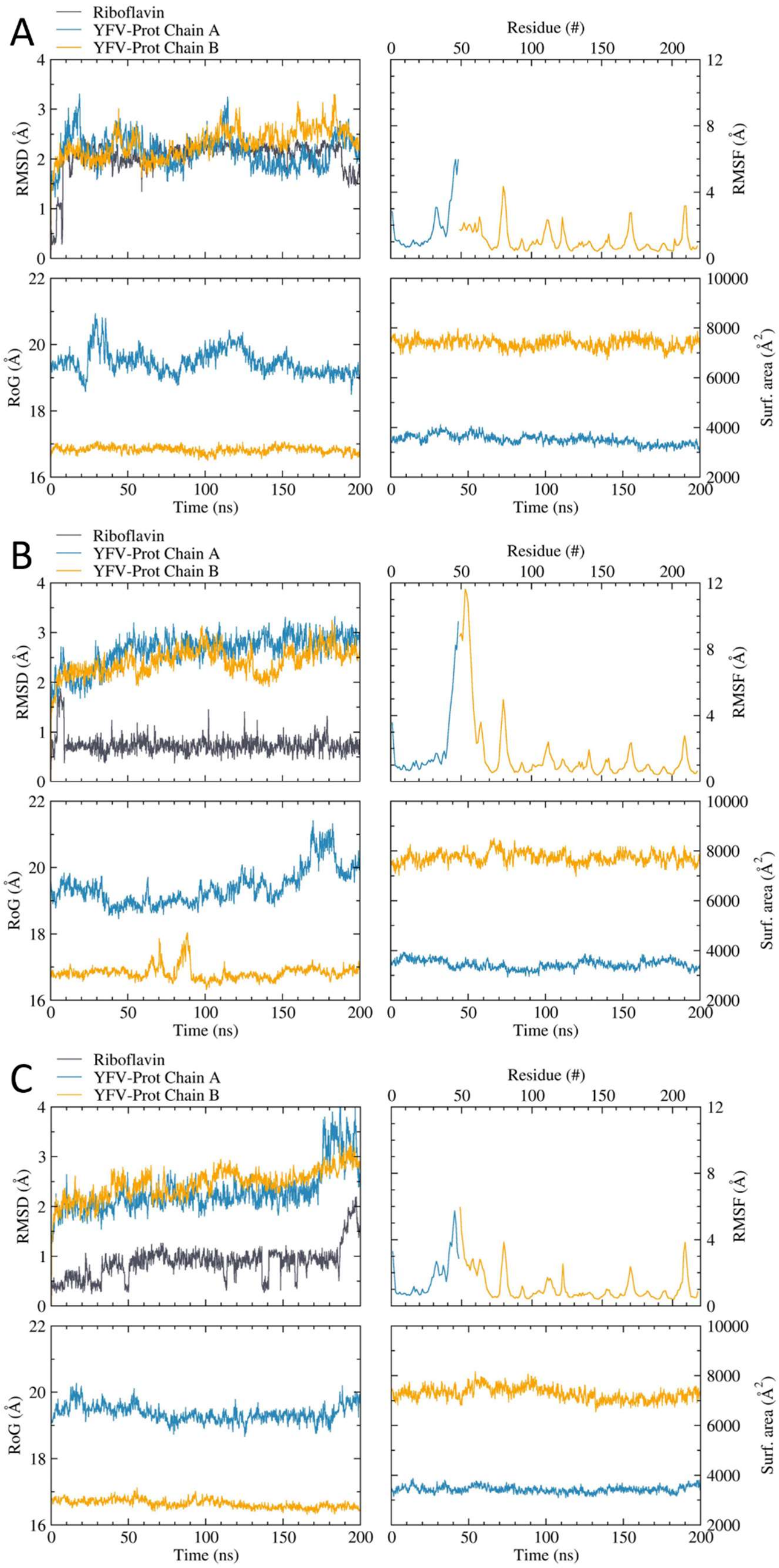

Figure S9. Time dependent modifications of the YFV NS2B/NS3^{pro}-riboflavin complex. NS2B (blue), NS3pro (orange) and riboflavin (dark grey). RMSD, RMSF, RoG and surface area as function of time. RMSF for each amino acid. **(A)** replicate one, **(B)** replicate two and **(C)** replicate three.

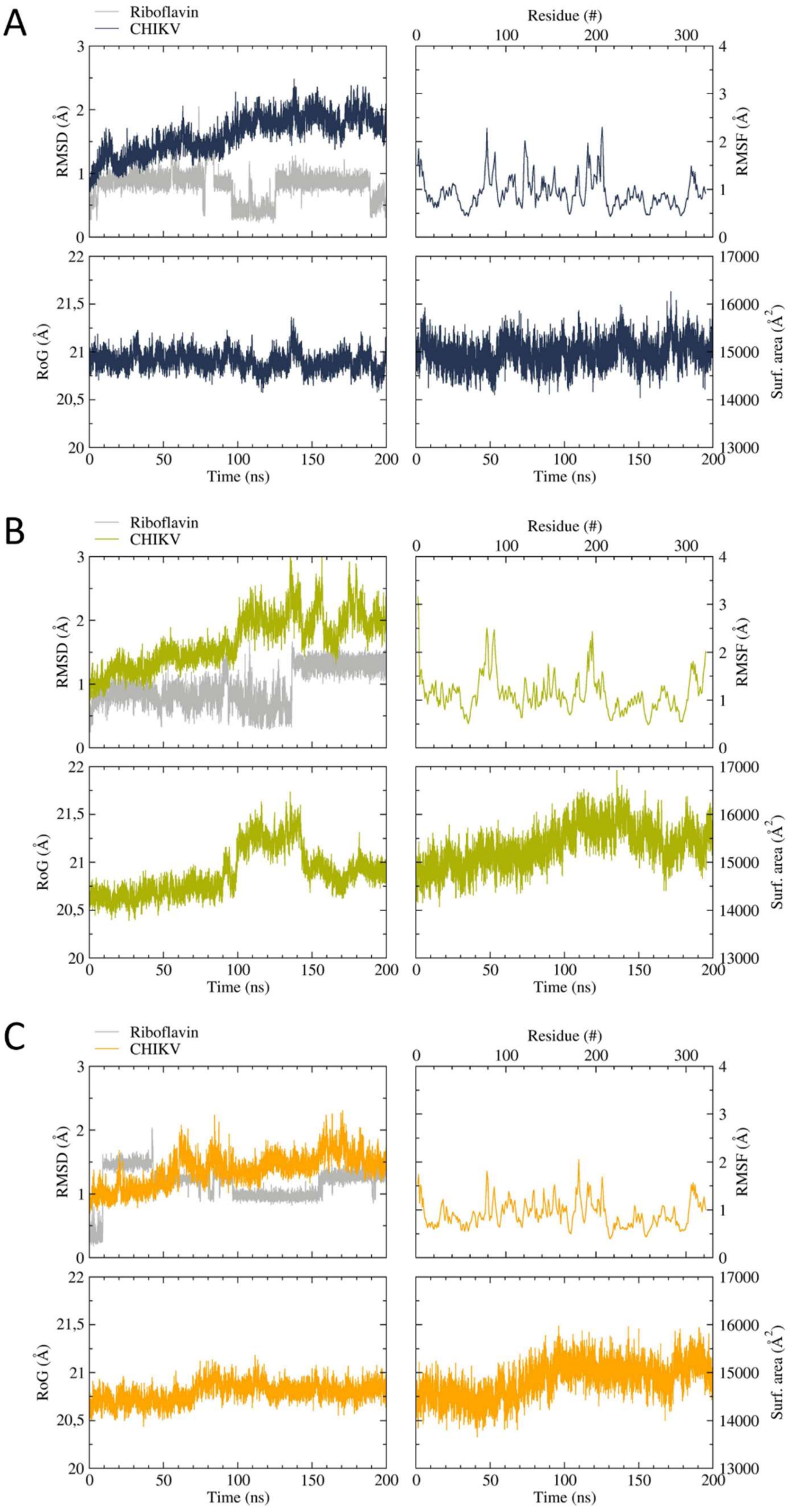

Figure S10. Time dependent modifications of the CHIKV $nsP2^{pro}$-riboflavin complex. CHIKV $nsP2^{pro}$ (dark grey (A), green (B) and orange (C)) and riboflavin (grey). RMSD, RMSF, RoG and surface area as function of time. RMSF for each amino acid. **(A)** replicate one, **(B)** replicate two and **(C)** replicate three.

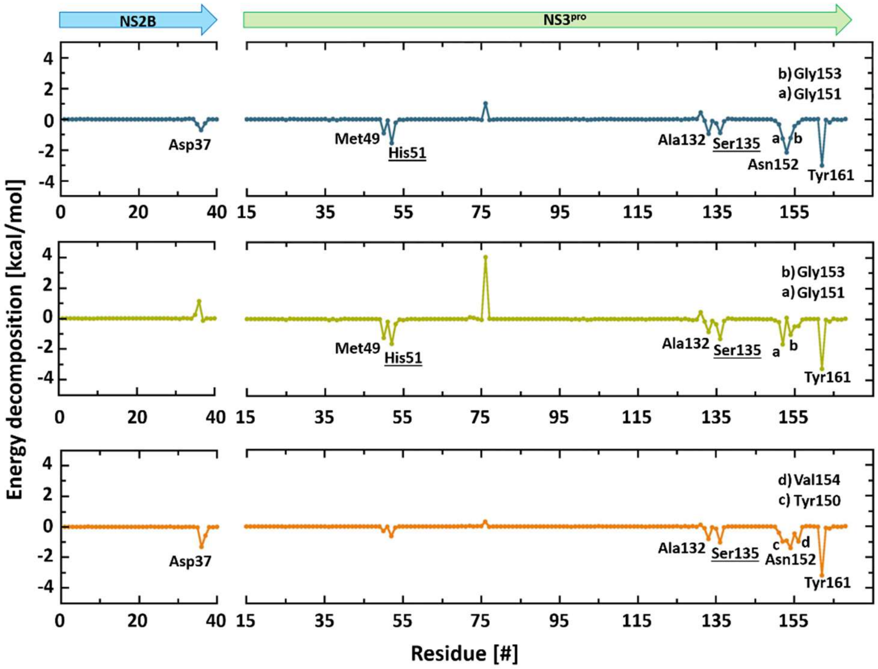

Figure S11. Decomposition of the binding energy of ZIKV NS2B/NS3^{pro}-riboflavin complex of three independent replicas. NS2B and NS3^{pro} are labelled by arrows. The amino acid residues involved in the interaction with riboflavin are labelled with name and sequence number.

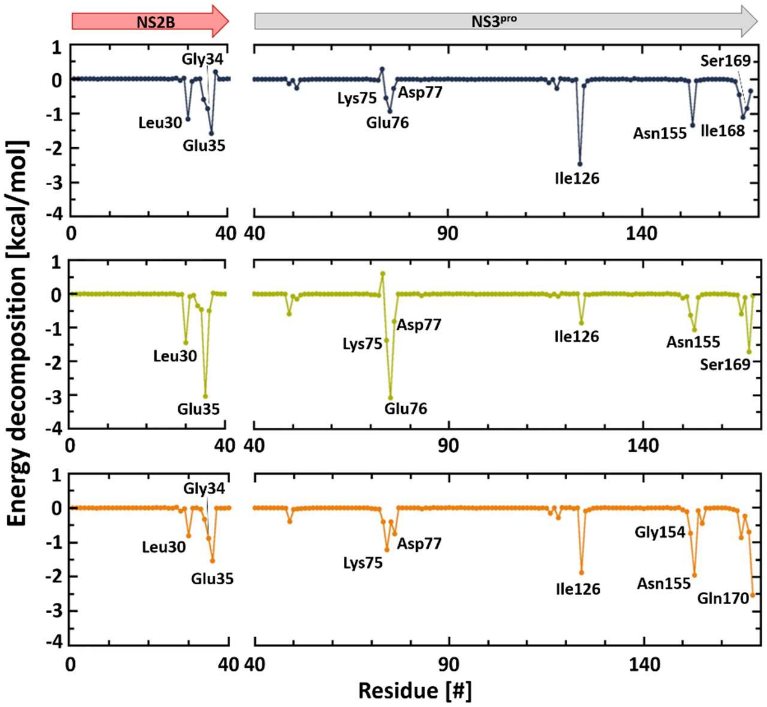

Figure S12. Decomposition of the binding energy of YFV NS2B/NS3^{pro}-riboflavin complex of three independent replicas. NS2B and NS3^{pro} are labelled and by arrows. The amino acid residues involved in the interaction with HST are labelled with name and sequence number.

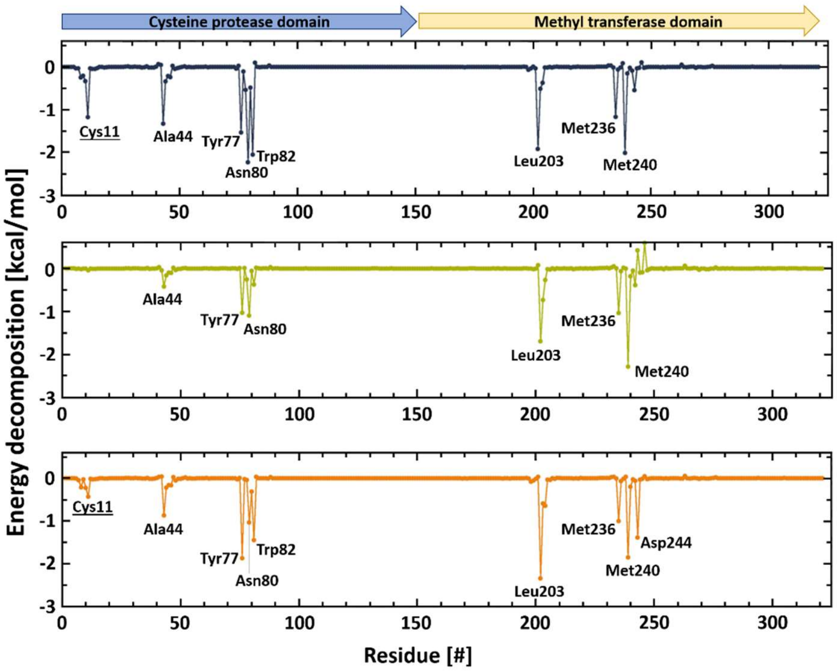

Figure S13. Decomposition of the binding energy of CHIKV nsP2^{pro}-riboflavin complex of three independent replicas. NS2B and NS3^{pro} are labelled and by arrows. The amino acid residues involved in the interaction with riboflavin are labelled with name and sequence number.

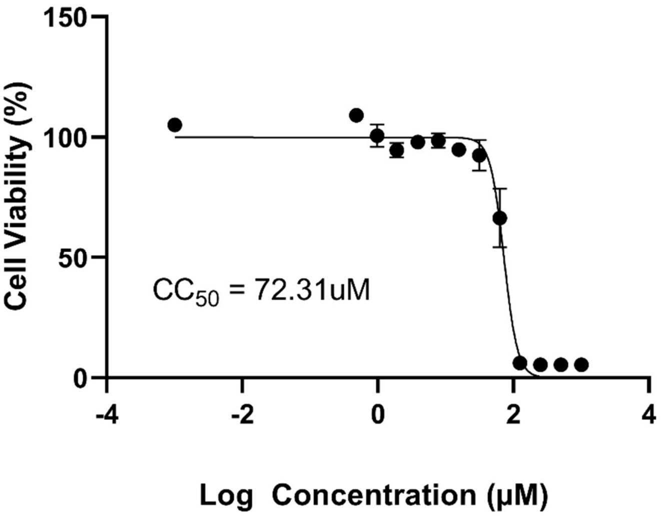

Figure S14. Cytoxicity assay of riboflavin against vero cells. Vero Cells were treated with concentrations of the molecule ranging from 1000 to 0.48 μM for 48h and the cytotoxic concentration of 50% (CC_{50}) were determined. Cellular viability measured using an MTT assay. Mean values of three independent experiments each measured in quadruplicate including the standard deviation are shown.

Table S1. Virus protease amino acid residues forming hydrogen bonds and hydrophobic interaction with riboflavin.

	Hydrogen Bonds			Hydrophobic interaction
Virus protease	Protease Residue/Atom (Donor/Aceptor)	Riboflavin atom/ (Donor/Aceptor)	Distance [Å]	
**ZIKV**	Asp37/OD1	O4	2.9	Gly36, Asp37, Met49, His51, Asp75, Ala132 Ser135, Gly151, Asn152, Gly153, Val155, Tyr161
	Ser135/OG	N3	2.9	
	Asn152/OD1	O4	3.0	
	Gly153/N	O5	3.1	
**YFV**	Glu35/OE2	O4	2.4	Gln33, Gly34, Glu35, His53, Lys75, Glu76, Asp77, Leu78, Gly154, Asn155, Ser169
	Asn155/N	O1	2.8	
	Ser169/OG	N3	2.9	
**CHIKV**	Tyr77/OH	O6	2.9	Ala44, Ser46, Tyr77, Asn80, Trp82, Leu203, Gln239, Met240, Asp244 Arg247
	Asn80/ND2	O1	3.0	
	Asn80/O	N3	3.1	
	Trp82/NE1	N4	3.1	
	Leu203/N	O3	2.9	

Table S2. Contributing amino acids in the Substrate-binding sites of ZIKV, YFV NS2B/NS3pro and CHIKV nsP2pro.

Substrate subsite	ZIKV NS2B/NS3pro residues	YFV NS2B/NS3pro residues	CHIKV nsP2pro residues
**S1`**	Val36		Ala8
	His51	His53	Asn9
	Ser135	Ser138	Cys11
			Lys14
			His81
**S1**	Tyr150	Asp132	Asn9
	Ser163	Tyr153	Cys11
	Ile165	Phe164	Trp12
			Asn80
			His81
**S2**	His51	His53	Trp12
	Gly151	Asp77	Tyr45
	Asn152	Leu78	Trp82
		Gly154	
		Asn155	
**S3**	Leu128	Leu131	Tyr77
	Asp129	Tyr133	Asn80
	Ala132		Met240
	Val155		
**S4**	Gly153	Ile157	Asn80
	Val154	Leu158	Trp82
	Val155		Gln239
			Asp244
**Oxyanion hole**	Ala132	Ser135	Ala8
	Gly133	Gly136	Asn9
	Thr134	Thr137	Val10
	Ser135	Ser138	Cys11
**References**	[1]	[2,3]	[4]

[1] Phoo WW, Li Y, Zhang Z, Lee MY, Loh YR, Tan YB, et al. Structure of the NS2B-NS3 protease from Zika virus after self-cleavage. Nat. Commun. 2016;7:1-8. https://doi.org/10.1038/ncomms13410

[2] Loehr, Kristina, John E. Knox, Wai Yee Phong, Ngai Ling Ma, Zheng Yin, Aruna Sampath, Sejal J. Patel et al. "Yellow fever virus NS3 protease: peptide-inhibition studies." Journal of general virology 88, no. 8 (2007): 2223-2227. https://doi.org/10.1099/vir.0.82735-0

[3] Noske, Gabriela Dias, Victor Oliveira Gawriljuk, Rafaela Sachetto Fernandes, Nathalia Dias Furtado, Myrna Cristina Bonaldo, Glaucius Oliva, and Andre Schutzer Godoy. "Structural characterization and polymorphism analysis of the NS2B-NS3 protease from the 2017 Brazilian circulating strain of Yellow Fever virus." Biochimica et Biophysica Acta (BBA)-General Subjects 1864, no. 4 (2020): 129521. https://doi.org/10.1016/j.bbagen.2020.129521

[4] Narwal M, Singh H, Pratap S. Crystal structure of chikungunya virus nsP2 cysteine protease reveals a putative flexible loop blocking its active site. Int. J. Biol. Macromol. 2018;116:451-462. https://doi.org/10.1016/j.ijbiomac.2018.05.007
